# *S*-acylation of the Insulin-Responsive Aminopeptidase (IRAP): Quantitative analysis and Identification of Modified Cysteines

**DOI:** 10.1038/srep12413

**Published:** 2015-07-22

**Authors:** Martin W. Werno, Luke H. Chamberlain

**Affiliations:** 1 Strathclyde Institute of Pharmacy and Biomedical Sciences, University of Strathclyde, 161 Cathedral Street, Glasgow G4 0RE, United Kingdom

## Abstract

The insulin-responsive aminopeptidase (IRAP) was recently identified as an *S*-acylated protein in adipocytes and other tissues. However, there is currently no information on the extent of *S*-acylation of this protein, the residues that are modified, or the effects of *S*-acylation on IRAP localisation. In this study, we employ a semi-quantitative acyl-RAC technique to show that approximately 60% of IRAP is *S*-acylated in 3T3-L1 adipocytes. In contrast, *S*-acylation of GLUT4, a glucose transporter that extensively co-localises with IRAP, was approximately five-fold lower. Site-directed mutagenesis was employed to map the sites of *S*-acylation on IRAP to two cysteine residues, one of which is predicted to lie in the cytoplasmic side of the single transmembrane domain and the other which is just upstream of this transmembrane domain; our results suggest that these cysteines may be modified in a mutually-exclusive manner. Although *S*-acylation regulates the intracellular trafficking of several transmembrane proteins, we did not detect any effects of mutating the modified cysteines on the plasma membrane localisation of IRAP in HEK293T cells, suggesting that *S*-acylation is not essential for the movement of IRAP through the secretory pathway.

Protein *S*-acylation (also referred to as palmitoylation) is a post-translational modification (PTM) involving the attachment of palmitate and other fatty acids to cysteine residues of proteins via thioester linkage. Numerous studies have demonstrated that *S*-acylation can mediate membrane attachment of soluble proteins, and regulate the intracellular trafficking of both soluble and transmembrane proteins and their partitioning into membrane micro-domains such as lipid rafts^1^. Furthermore, *S*-acylation can also affect protein-protein interactions and protein stability^2^.

*S*-acylation is mediated by a family of membrane-bound enzymes comprising 24 isoforms in mammals^3^,^4^. These palmitoyl-transferases (PATs) are referred to as zDHHC enzymes due to the presence of a conserved DHHC (Asp-His-His-Cys) amino acid motif present within a zinc-finger cysteine-rich domain^5^,^6^,^7^. Unlike other lipid modifications, *S*-acylation is reversible and dynamic due to the labile nature of the thioester bond^8^. Depalmitoylation is catalysed by enzymes including acyl-protein thioesterases (APTs)^9^, and cycles of *S*-acylation and deacylation play an important role in regulating dynamic changes in protein localisation, often in response to specific signals^10^,^11^.

Insulin-stimulated translocation of GLUT4 storage vesicles (GSVs) to the plasma membrane in adipocytes and skeletal muscle cells is essential for blood glucose homeostasis^12^, and defects in this pathway are linked to obesity and diabetes^13^,^14^. The insulin-stimulated translocation of GLUT4 to the plasma membrane is regulated by various PTMs, most notably phosphorylation. In addition, some studies have also highlighted the potential involvement of *S*-acylation in these pathways. A variety of important proteins including the insulin receptor and the SNARE protein SNAP23 have long been known to be *S*-acylated^15^,^16^. More recent studies identified additional targets of the *S*-acylation machinery in adipocytes, including GLUT4 and the insulin-responsive aminopeptidase (IRAP)^17^, a protein that co-localises extensively with GLUT4 and regulates the formation and/or trafficking of insulin-responsive GLUT4 storage vesicles^18^. *S*-acylation was recently suggested to occur at a single site on GLUT4 (Cys223) and this modification may be involved in the sorting of GLUT4 to insulin-responsive vesicles^19^. Unlike GLUT4, which has a highly restricted expression to adipocytes and skeletal and cardiac muscle, IRAP is expressed widely, including in the brain^20^. At present, the sites of *S*-acylation on IRAP or how this modification affects the localisation of the protein are not known. Furthermore, there is no information on the likely extent of *S*-acylation of IRAP and how this compares with proteins such as GLUT4.

## Results

### Quantitative analysis of IRAP S-acylation in 3T3-L1 adipocytes

**Figure 1:**
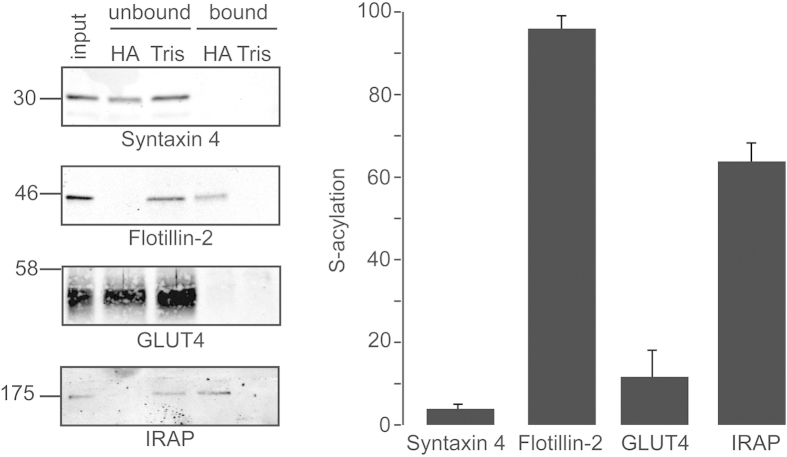
Semi-quantitative analysis of IRAP *S*-acylation in 3T3-L1 adipocytes. On day 9 following initiation of the cell differentiation protocol, 3T3-L1 cells were incubated overnight in media containing 5.55 mM D-glucose. Cell membranes were then recovered by centrifugation and incubated with MMTS (1.5% v/v), and then with either 0.5 M hydroxylamine (HA) or 0.5 M Tris together with free thiol group binding beads. *Left panel:* Recovered fractions were analysed by immunoblotting with the indicated antibodies. Position of molecular weight standards are shown on the left. Equivalent amounts of unbound and bound fractions were loaded, whereas the input sample was loaded at half this amount. *Right panel:* Quantification of *S*-acylation levels of proteins was determined using densitometry of immunoblots shown in panel A (n = 3).

To generate information on the extent of IRAP *S*-acylation, we optimised acyl-RAC (resin-assisted capture) to allow the percentage *S*-acylation of proteins to be estimated by comparing immunoreactivity in equivalent amounts of unbound and bound fractions. Differentiated 3T3-L1 adipocytes were incubated overnight in medium containing 5.55 mM D-Glucose, and subsequently total membrane proteins were isolated and subjected to acyl-RAC to enrich *S*-acylated proteins^21^. This procedure allows the capture of *S*-acylated proteins on thioreactive sepharose following the removal of *S*-acyl chains by 0.5 M hydroxylamine (HA) treatment. As a control, membrane fractions in parallel samples were treated with 0.5 M Tris. When performing acyl-RAC, we recovered bound and unbound fractions in equal volumes and resolved the same volume of each fraction by SDS-PAGE. This enabled a semi-quantitative assessment of the percentage S-acylation of specific proteins that were detected by immunoblotting. As controls, we analysed Flotillin-2, a known *S*-acylated protein^22^, and Syntaxin 4, a transmembrane protein that has not been reported to be modified by *S*-acylation. Whereas ~96% of Flotillin-2 was estimated to be *S*-acylated by applying this method, the fraction of syntaxin 4 recovered as an *S*-acylated protein was only 3.9% (Fig. 1). Analysis of IRAP immunoreactivity in the recovered fractions was consistent with ~60% of this protein being *S*-acylated, whereas the level of *S*-acylation of GLUT4 was estimated to be less than 12% (Fig. 1).

### IRAP is *S*-acylated on two cysteine residues positioned in and around the single transmembrane domain

**Figure 2:**
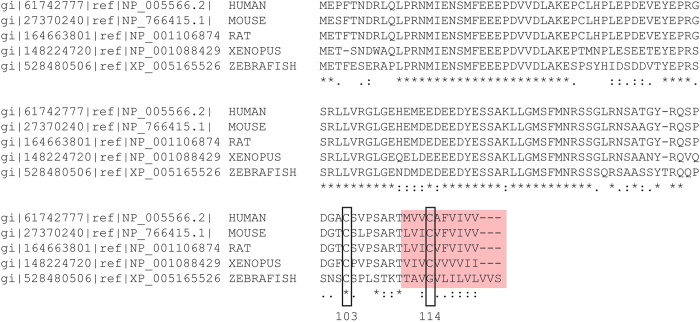
Alignment of the N-terminal sequences of IRAP from human, mouse, rat, Xenopus and zebrafish. Identical amino acids are highlighted by *asterisks*, similar amino acids are highlighted by *dots*. Cysteine-103 and cysteine-114 are highlighted by *boxes*. The amino acids predicted to form the start of the transmembrane domain are highlighted by red shading.

**Figure 3:**
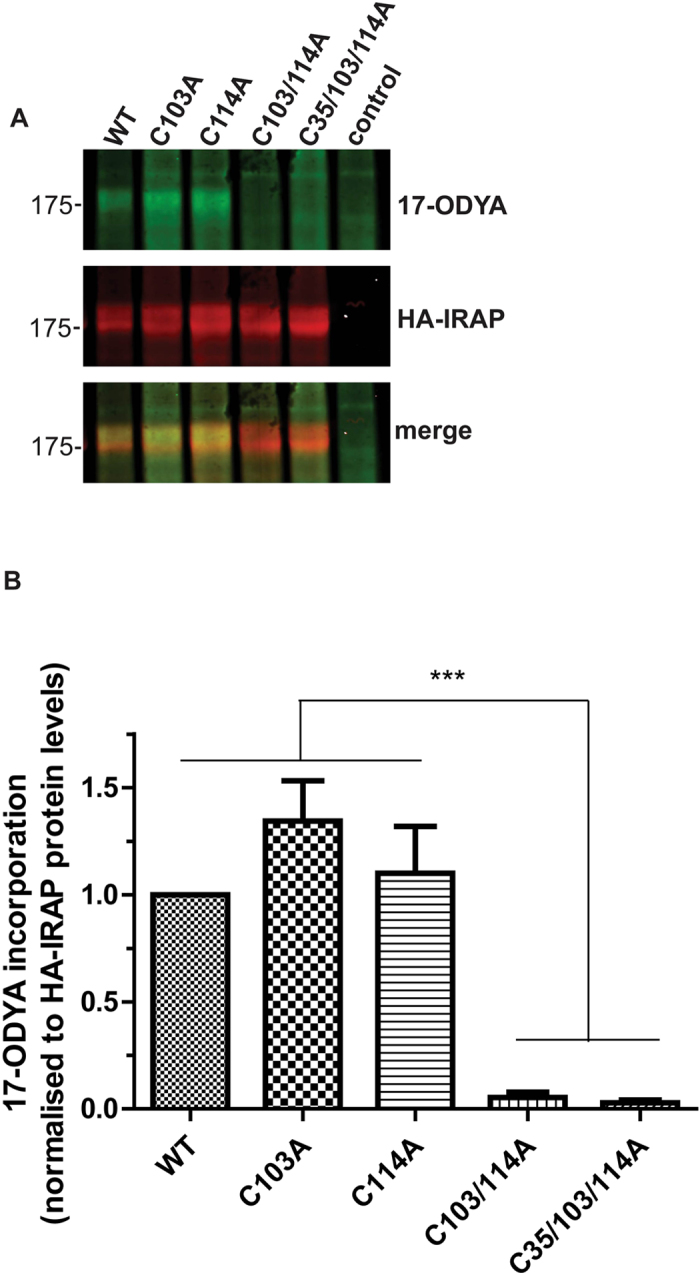
Mapping of *S*-acylation sites in IRAP. HEK293T cells were transfected with plasmids encoding the indicated HA-tagged IRAP constructs for 24 h. Cells were then metabolically labelled with 15 μM 17-ODYA and incorporation of 17-ODYA into IRAP was detected by reaction with IRDye 800CW azide. Samples were subjected to SDS-PAGE and analysed by immunoblotting. Anti-HA antibody binding and IRDye were visualised using a LICOR Odyssey infrared imaging system. **A** Representative images showing 17-ODYA incorporation into wild-type and mutant IRAP (*top panel*), HA immunoreactivity (*middle panel*) and a merge (*bottom panel*). Position of molecular weight markers are shown on the left. **B** Quantification of 17-ODYA incorporation into IRAP constructs. Quantified values of the 17ODYA signal from IRAP constructs were normalised to protein expression level. The calculated value of 17-ODYA incorporation into wild type IRAP was set to 1 and all mutants were expressed relative to this. One way ANOVA followed by Tukey’s multiple comparison test was applied for statistical analysis. ***P ≤ 0.001 (n = 5).

IRAP contains 1,025 amino acids but only the N-terminal 110 amino acids are cytoplasmic, with a single transmembrane domain (TMD) predicted to start at amino acid 109/110 (mouse/human sequences; Uniprot). Integral membrane proteins are often *S*-acylated at cysteines present at a juxtamembrane position or in the cytosolic end of transmembrane sequences. Two cysteines were of particular interest as potential *S*-acylation sites: Cys-103 and Cys-114, both of which are highly conserved and present in or around the TMD (Fig. 2). To investigate if these residues are important for *S*-acylation of IRAP, we generated an HA-tagged form of the protein and transfected this or the following cysteine mutants into HEK293T cells: C103A, C114A, C103A/C114A and C35A/C103A/C114A. The C35A/C103A/C114A triple mutant was included in the analysis as it lacks any cysteines in the cytoplasmic region of the protein. HEK293T cells were used rather than 3T3-L1 adipocytes as they are more amenable to transfection and *S*-acylation analysis. Combined mutation of both C103A and C114A led to a complete loss of IRAP *S*-acylation, as detected using click chemistry with 17-ODYA (Fig. 3). Interestingly however, individual mutation of either Cys-103 or Cys-114 had little effect on the amount of labelling of IRAP by 17-ODYA. The control sample (non-transfected cells) confirmed the identity of the bands detected by the HA antibody.

### Mutation of the *S*-acylation sites in IRAP does not prevent plasma membrane targeting in HEK293T cells

**Figure 4:**
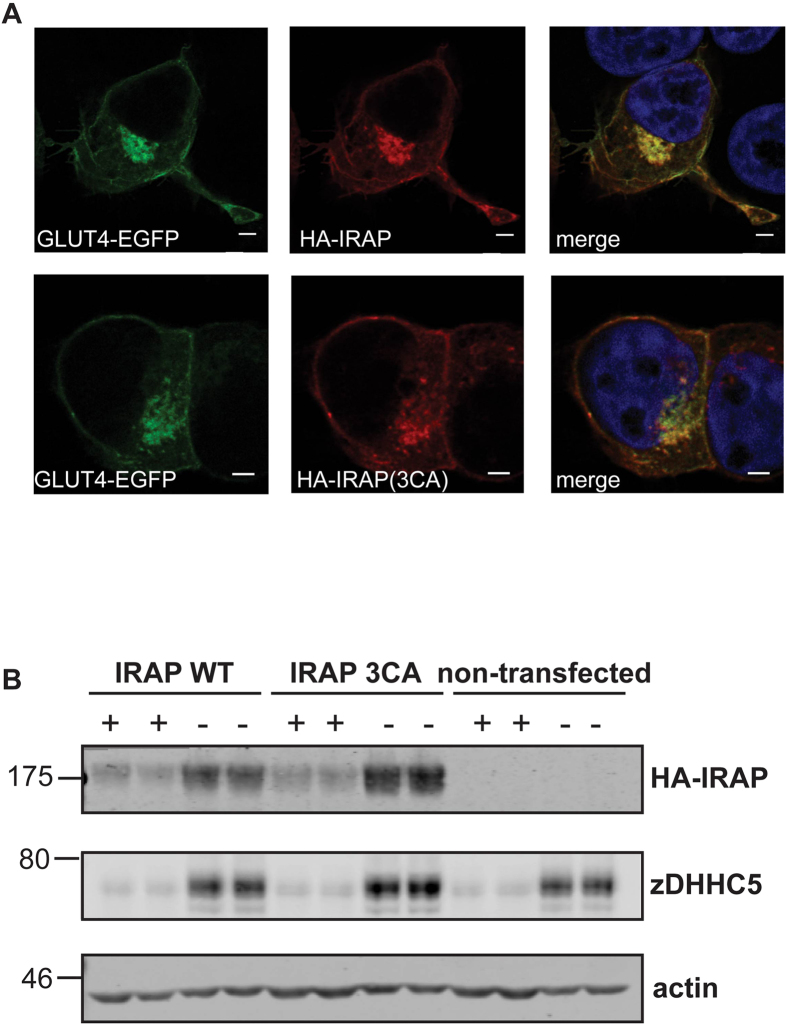
Intracellular localisation of wild-type and cysteine mutant IRAP. **A** HEK293T cells were co-transfected with HA-tagged IRAP constructs together with GLUT4-EGFP. After 24 h, cells were fixed in 4% formaldehyde and subsequently permeabilised with Triton X-100. Cells were then incubated in HA antibody and subsequently in secondary antibody conjugated to Alexa Fluor 543. Cells were mounted in ProLong Gold Antifade mounting medium containing DAPI. Whole cell image stacks were acquired by confocal microscopy. Representative sections of a typical cell are shown. Separate channels displaying GLUT4-EGFP (green) and HA-tagged IRAP WT or 3CA mutant (red) and a merge of both channels including DAPI staining are shown. Scale bar = 5 μm. **B** Cells were transfected for 24 h with plasmids encoding HA-tagged IRAP wild type or cysteine to alanine mutant (3CA) and were incubated in trypsin-EDTA (0.05%) (+) or in standard culture media (–) for 30 minutes. Cell lysates were subjected to SDS-PAGE followed by immunoblotting with the indicated antibodies. Anti-HA was used for detection of HA-IRAP. Samples were loaded in duplicate and representative immunoblots are shown, with the position of molecular weight markers indicated on the left hand side of blots.

*S*-acylation can modulate the intracellular targeting of both transmembrane proteins and soluble proteins. To investigate the effect of *S*-acylation on the trafficking of IRAP, we compared the localisation of wild-type and 3CA mutant (C35A/C103A/C114A). These HA-tagged IRAP constructs were co-transfected into HEK293T cells with GLUT4-EGFP to provide a co-localising reference protein. As shown in Fig. 4A, there was no obvious difference in the localisation of wild-type or 3CA mutant IRAP relative to GLUT4-EGFP, and both IRAP proteins showed detectable staining of the plasma membrane. To compare more closely the plasma membrane localisation of wild-type and 3CA mutant IRAP in cell populations, transfected cells were treated with trypsin to digest cell surface-exposed proteins. To test for the expected effects of trypsin, we examined endogenous zDHHC5, a plasma membrane enzyme^23^. Figure 4B shows that trypsin treatment led to a loss of zDHHC5 immunoreactivity, whereas little effect was observed on actin levels (confirming that trypsin does not enter cells). Trypsin treatment also led to a marked loss in immunoreactivity of both wild-type and mutant IRAP (Fig. 4B), implying that both proteins are expressed at the plasma membrane at similar levels, in agreement with the immunofluorescence analysis (Fig. 4A).

## Discussion

The high level of constitutive *S*-acylation of IRAP (~60% by our analysis) suggests that this modification is likely to be important for a fundamental property of the protein. Interestingly, a smaller pool of GLUT4 was estimated to be *S*-acylated (<12%). As *S*-acylation has been suggested to regulate the intracellular trafficking of GLUT4^19^, it will be interesting in follow-up work to investigate if this modification is differentially regulated on GLUT4 and IRAP. For example, if the *S*-acylation of GLUT4 is highly reversible then this may account for the lower levels of this protein recovered in acyl-RAC fractions. It is also possible that the existence of *S*-acylated and non-acylated pools of GLUT4 is linked to different subcellular pools of the protein, and this also merits further investigation. Notwithstanding these issues, the marked difference in the estimated *S*-acylation levels of two proteins that co-localise extensively in adipocytes highlights the importance of optimising acyl-RAC or acyl-biotin exchange (ABE) experiments to allow an assessment to be made of the likely extent of S-acylation of proteins of interest. A potential caveat relating to the quantitative aspect of this work is that we compared unbound and bound fractions and it is possible that some protein is lost during the wash steps of the acyl-RAC procedure. We preferred to compare the bound fraction to the unbound fraction for the quantitative analysis as these samples have been subjected to the same buffer conditions and overnight incubation conditions (see Methods). Interestingly, Ren *et al*.^17^ suggested that IRAP *S*-acylation was likely to be higher than *S*-acylation of Munc18c based on a qualitative comparison of bound and input samples. This suggestion is consistent with the observations made in the current study.

The double cysteine mutant (C103/114A) of IRAP displayed a near complete loss of incorporation of 17-ODYA but individual mutation of the cysteines had little effect on incorporation (Fig. 3). Hence, although the cysteine residue pair consisting of cysteine-103 and cysteine-114 is essential for IRAP *S*-acylation, there appears to be a complex relationship between these residues. We suggest that IRAP might be constitutively *S*-acylated on either Cys-103 or Cys-114 in a mutually exclusive manner. *S*-acylation at a single site might modify the structure or membrane topology of IRAP, preventing modification of the other cysteine. Alternatively, both cysteines might be *S*-acylated in the wild-type protein and mutation of one of these sites might make the other site more accessible to its respective zDHHC enzyme, increasing the rate of *S*-acylation at that site. Another possibility is that mutation of individual cysteines decreases the rate of deacylation of the other cysteine. Interestingly, a similar observation was made for the lipoprotein receptor-related proteins 6 (LRP6), which shares a common membrane topology to IRAP with a single TMD and two cysteines in proximity to the TMD. Simultaneous replacement of both of these cysteines in LRP6 to alanine resulted in a substantial loss of *S*-acylation, but individual cysteine replacement had almost no effect^24^.

At present, we do not know how *S*-acylation exerts regulation on IRAP. This modification did not appear to be required for plasma membrane targeting of the protein in HEK293T cells or for co-localisation with GLUT4 in these cells. IRAP is widely expressed and we also detected similar levels of *S*-acylation of the protein in rat brain. Thus, the analysis of IRAP localisation in HEK293T cells is valid. Nevertheless, it would also be of interest to examine if there is a specific requirement for *S*-acylation in the targeting of IRAP to insulin-responsive compartments in adipocyte, as was suggested recently for GLUT4^19^. Such a role would place *S*-acylation (and the enzymes that control this process) as an important factor required for efficient formation/trafficking of GLUT4 storage vesicles.

## Methods

### cDNA constructs

A plasmid containing mouse IRAP cDNA was synthesised and supplied by GeneArt®. This cDNA included a sequence encoding a triple HA-tag upstream of the IRAP coding sequence. This HA-IRAP construct was flanked by *SalI* and *BamHI* restriction sites to facilitate cloning into the EGFP-N1 vector (the presence of a stop codon in IRAP prevented translation of the downstream EGFP sequence). Cysteine residues at positions 35, 103 and 114 were mutated to alanine individually or in combination by site-directed mutagenesis.

GLUT4 was amplified from 3T3-L1 adipocyte cDNA and cloned into pEGFP-N1 using *HindIII* and *SalI* restriction sites.

### Antibodies

Monoclonal Flotillin-2 and monoclonal IRAP antibodies were obtained from Cell Signalling (MA, USA). GLUT4 antibody was from Abcam (Cambridge, UK). Anti-Syntaxin 4 rabbit polyclonal antibody was purchased from Synaptic Systems (Goettingen, Germany). zDHHC5 antibody was from Sigma (Poole, UK). Anti-HA rat monoclonal antibody was purchased from Roche (Basel, Switzerland).

### Cell culture

Mouse 3T3-L1 adipocytes were cultured in Dulbecco’s Modified Eagle Medium (DMEM) GlutaMAX™, supplemented with 10% newborn calf serum and 1% penicillin/streptomycin. Controlled conditions were maintained in a humidified cell incubator set to a temperature of 37 ^O^C and containing 10% CO_2_. The differentiation of 3T3-L1 cells into adipocytes was induced by adding differentiation media consisting of DMEM GlutaMAX™ containing 10% fetal bovine serum, 1% penicillin/streptomycin, insulin (170 nM), troglitazone (1 μM), 3-isobutyl-1-methylxanthine (IBMX) (500 μM) and dexamethasone (0.25 μM) to pre-adipocytes that had been confluent for approximately 48 h. After three days the media was replaced with differentiation media without IBMX and dexamethasone. Adipocytes were used 9 days following the start of the differentiation protocol.

HEK293T cells were cultured in DMEM containing 10% fetal bovine serum in a humidified incubator at 37 °C and 5% CO_2_. Cells were transfected using Lipofectamine 2000 according to the manufacturer’s instructions (Life Technologies).

### Acyl-RAC

Prior to harvesting, 3T3-L1 adipocytes were incubated overnight in medium containing 5.55 mM D-Glucose. Subsequently, cells were collected in buffer A (25 mM HEPES, 25 mM NaCl, 1 mM EDTA, pH 7.4 and protease inhibitor cocktail) and passed five to ten times through a 26G needle. Following disruption, the cell fraction was centrifuged at 800 × g for 5 minutes at 4 °C, and the recovered supernatant was then subjected to an additional centrifugation step at 136,000 × g for 60 min at 4 °C. The pellet containing the membrane fraction was then resuspended in 100 μl buffer A containing 0.5% Triton X-100 (v/v). In order to block free SH groups with S-methyl methanethiosulfonate (MMTS), 200 μl of blocking buffer (100 mM HEPES, 1 mM EDTA, 87.5 mM SDS and 1–1.5% (v/v) MMTS) was added to the resuspended proteins and incubated for 4 h at 40 °C with frequent vortexing. Subsequently, 3 volumes of ice-cold 100% acetone was added to the blocking protein mixture and incubated for 20 minutes at −20 °C and then centrifuged at 5,000 × g for 10 minutes at 4 °C to pellet precipitated proteins. The pellet was washed five times in 1 ml of 70% (v/v) acetone and resuspended in buffer B (100 mM HEPES, 1 mM EDTA, 35 mM SDS). A fraction of the solubilised pellet was saved as the input. For treatment with hydroxylamine (HA) and capture by Thiopropyl Sepharose® beads, 2 M HA was added together with the beads (previously activated for 15 min with dH_2_O) to a final concentration of 0.5 M HA and 10% (w/v) beads. As a negative control, 2 M Tris was used instead of HA. These samples were then incubated overnight at room temperature with end-over-end mixing. The supernatant was removed and retained as the “unbound” fraction. The remaining beads were washed five times with 1 ml buffer B. Subsequently, the proteins were eluted from the beads by two consecutive incubations in 100 μl SDS sample buffer containing 50 mM dithiothreitol (DTT) for 15 minutes at room temperature and then 5 minutes at 95 °C. The eluted proteins were the “bound” fraction. Before subjecting all samples to SDS-PAGE, the final volumes of the bound and unbound fractions were equalised. For quantification of *S*-acylation, we used the following equation: [Bound(HA)/(Bound(HA) + Unbound(HA)] − [Bound (Tris)/(Bound(Tris) + Unbound(Tris)].

### Metabolic labelling and click chemistry

HEK293T on 24-well plates were transfected with 1 μg plasmid per well. 24 h post-transfection, cells were incubated in serum-free medium containing 1% (w/v) fatty acid-free bovine serum albumin (BSA) for 30 minutes. For metabolic labelling, cells were incubated in serum-free culture medium containing 1% (w/v) fatty acid-free BSA and 15 μM 17ODYA for 4 hours. All incubations of the cells were carried out in a cell culture incubator with saturated humidity, 5% CO_2_ and at 37^o^C. Subsequently, cells were lysed in 100 μl of click-chemistry lysis buffer (50 mM Tris, 17 mM SDS, pH 8.0 containing protease inhibitors). For the click-reaction, 80 μl of click-reaction mix (5 mM CuSO4, 500 μM Tris(benzyltriazolylmethyl)amine (TBTA), 25 μM IRDye 800 CW azide) was added to the lysed cells and vortexed. This was then immediately supplemented with 20 μl of ascorbic acid (4 mM) and incubated for one hour at room temperature with end-over-end mixing. Following the click-reaction three volumes of ice-cold 100% acetone was added and incubated at −20 °C for 20 minutes. Precipitated proteins were pelleted by centrifugation for 5 minutes at 13,000 x g and 4 °C, washed 3 times in 70% ice-cold acetone and centrifuged each time for 5 minutes at 13,000 × g at 4 °C. The pellet was then dissolved in 100 μl SDS sample buffer with 25 mM DTT and heated for 5 minutes at 95 °C. The samples were then analysed by SDS-PAGE and immunoblotting.

### Trypsin digestion of surface-exposed membrane proteins

HEK293T cells transfected for 24 h with plasmids encoding the protein of interest (1 μg/well of a 6-well plate) were washed with PBS, and then incubated in trypsin-EDTA solution (0.05%) or HEK293T culture media (control) for 30 min in a cell culture incubator at 37 °C/5% CO_2_. Subsequently, the cells were transferred into eppendorf tubes and pelleted by centrifugation. The cell pellets were then washed twice by adding 1 ml PBS followed by centrifugation, resuspended in 200 μl SDS sample buffer with 25 mM DTT and subjected to SDS-PAGE followed by immunoblotting.

### Confocal microscopy

Transfected cells were washed twice with PBS and fixed in 4% (v/v) of formaldehyde for 30 minutes at room temperature. The fixed cells were washed in PBS containing 0.3% BSA (PBS-BSA) and then permeabilised in PBS-BSA containing 0.25% Triton X-100 for 10 min. Permeabilised cells were washed in PBS-BSA and incubated in HA antibody (1:50 in PBS-BSA) for 60 min. The cells were washed again in PBS-BSA to remove free HA antibody and then incubated for 60 min in PBS-BSA containing anti-mouse antibody conjugated to Alexa Fluor 543 dye (1:400). The cells were then washed in PBS, air-dried and mounted onto microscope slides using ProLong Gold Antifade Reagent with DAPI (4’,6-Diamidino-2-Phenylindole) mounting medium (Life Technologies (Paisley, U.K.)). A Leica SP5 Confocal Laser Scanning Microscope was used for image acquisition. Image stacks were deconvolved using Huygen’s software.

### Data analysis

The software Image Studio™ Lite V3.1 (LI-COR Biosciences, Lincoln, NE, U.S.A.) was used for quantification of immunoblots. The mean of the measured values was presented with the standard error of the mean (SEM). To assess statistical significance, a one-way analysis of variance (ANOVA) in conjunction with a Tukey’s Test was performed (GraphPad Prism® software).

## Additional Information

**How to cite this article**: Werno, M.W. and Chamberlain, L.H. *S*-acylation of the Insulin-Responsive Aminopeptidase (IRAP): Quantitative analysis and Identification of Modified Cysteines. *Sci. Rep*. **5**, 12413; doi: 10.1038/srep12413 (2015).
